# Transition Metal Complexes of Quinolino[3,2-b]benzodiazepine and Quinolino[3,2-b]benzoxazepine: Synthesis, Characterization, and Antimicrobial Studies

**DOI:** 10.1155/2007/42587

**Published:** 2007-09-19

**Authors:** B. Basavaraju, Halehatty S. Bhojya Naik, Mustur C. Prabhakara

**Affiliations:** ^1^Department of Biotechnology, GM Institute of Technology, Davangere 577 006, Karnataka, India; ^2^Department of PG Studies and Research in Industrial Chemistry, School of Chemical Sciences, Kuvempu University, Shankaraghatta 577 451, Shimoga, Karnataka, India

## Abstract

The synthesis and characterization of title complexes of the ligand Quinolino[3,2-b]benzodiazepine (QBD) and Quinolino[3,2-b]benzoxazepine (QBO) are reported. The complexes have been characterized by elemental analysis, molar conductance, magnetic studies, IR, H1 NMR, and UV-visible studies. They have the stoichiometry [${\text{ML}}_{2}\text{C}1_{2}$], where M=Co(II)/Ni(II), L=QBD/QBO, and [$\text{MLC}1_{2}$], where M=Zn(II)/Cd(II), L=QBD/QBO. The antibacterial and antifungal activity of the metal complexes has been investigated. The complexes were found to have higher antimicrobial activity than the parent ligand.

## 1. INTRODUCTION

Quinoline derivatives represent the major class of heterocycles, and a number of preparations have been known since the late 1980s. The quinoline skeleton is often used for the design of many synthetic compounds with diverse pharmacological properties. Dynemicin A and Streptonigrin are naturally occurring members of the class of antitumor antibiotics, whose syntheses are based on the utilization of preformed quinoline derivatives [1]. The 8-(diethylaminohexylamino)-6-methoxy-4-methyl-quinoline is highly effective against the protozoan parasite *Trypanosoma cruzi*, which is the agent of Chagas disease [2] and the 2-(2-methylquinolin-4-ylamino)-N-phenylacetamide is more active than the standard antileishmanial drug sodium antimony gluconate [3]. The centipede, *Scolopendra Subspinipes mutilalns* L. KOCH, which is found to contain 3,8-dihydroxyquinoline called *Jineol* has been prescribed for tetanus and childhood convulsions [4]. This drug has also been used for many other clinical purposes, such as the treatment of acute heart attack and as a toxicide in Korea [5]. Cryptolepine (5-methyl-5H-indolol[3,2-b]quinoline)-major *Cryptolepis sanguinolenta* alkaloid displays a plenty of pharmacological effects, such as antimuscarinic, noradrenergic receptor antagonistic, antihypertensive, vasodilative, antithrombotic, antipyretic, and anti-inflammatory properties. Neocryptolepine and cryptolepine derivatives reveal antiplasmodial, antitrypanosomal, and first of all, cytotoxic activities [6–8]. Quinoline containing drugs, particularly 4-aminoquinolines, have a long and successful history as antimalarials [9, 10].

## 2. MATERIALS AND METHODS

### 2.1. Analytical methods

All the chemicals used in the present study are of AR grade. 2-Chloro-3-quinolinecarbaldehyde (Sigma-Aldrich Chemie, Germany), 2-aminophenol (S.D. Fine Chem Ltd, India), and *o*-Phenylenediammine (S.D. Fine Chem Ltd, India) were used. The metal contents of the complexes were determined by complexometric titrations and gravimetric estimations. The molar conductivities in DMF (10^−3^ M) at room temperature were measured using an Equiptronics digital conductivity meter. Magnetic susceptibilities of the solid complexes were measured employing Gouy balance at room temperature (28 ± 2°C) using Hg[Co(CNS)_4_], that is, mercury(II) tetrathiocyanato cobaltate(II), as a calibrant for standardizing the Gouy tube.

### 2.2. Spectral measurements

The IR spectra of ligand and its metal complexes were recorded on a Shimadzu FTIR-8400S spectrometer with KBr pellets in the region 250–4000 cm^−1^. JEOL 60 MHz spectrometer was used for recording the proton NMR spectra employing TMS as internal reference and DMSO-d_6_ as solvent. UV-visible spectra were measured on a Shimadzu double beam spectrophotometer using N,N*′*-dimethylformamide as a solvent at 10^−3^ M concentration.

## 3. EXPERIMENTAL

### 3.1. Synthesis of ligand QBD by microwave irradiation

**Scheme 1:**
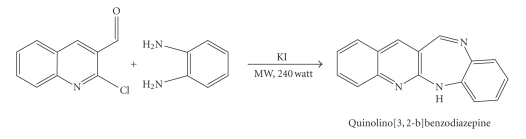
Preparation of Quinolino[3,2-b]benzodiazepine(QBD).

2-Chloro-3-quinolinecarbaldehyde (0.958 g, 5 mmo1) dissolved in small amount of acetic acid was taken in a 100 ml borosil beaker. *o*-Phenylenediammine (0.541 g, 5 mmol) and a pinch of potassium iodide were then added. The mixture was irradiated in a microwave oven for about 10 minutes. The product obtained was poured into ice-cold water, the solid separated was filtered, dried, and recrystallized (Scheme 1).

### 3.2. Synthesis of ligand QBO by microwave irradiation

**Scheme 2:**
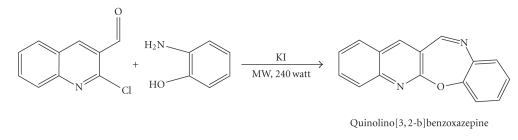
Preparation of Quinolino[3,2-b]benzoxazepine(QBO).

Mixture of 2-aminophenol (0.11 g, 1 mmol), KOH (0.057 g, 1 mmol), and 2 ml of DMSO were taken in a 100 ml borosil beaker. 2-Chloro-3-quinolinecarbaldehyde (1 mmol, 0.192 g) and a pinch of KI were then added. The mixture was irradiated for about two minutes in a microwave oven. The product obtained was then hydrolyzed by pouring into ice-cold water. The final product separated as a solid on acidification with dilute HCl was then filtered and dried (Scheme 2).

### 3.3. Synthesis of Cobalt(II) and Nickel(II) complexes of QBD and QBO

The ligand (10 mmol) was dissolved in a dry methanol (50 ml). A solution of the metal chloride (5 mmol) in methanol (50 ml) was then added dropwise to the ligand solution under nitrogen gas with continuous stirring. The mixture was refluxed for about 5 hours. The resulting precipitate was filtered off and washed with methanol. The precipitate was redissolved in ethanol on a water bath and the ethanol was slowly evaporated. Further, slow evaporation of the solution at room temperature resulted in the formation of colored compound.

### 3.4. Synthesis of Cadmium(II) and Zinc(II) complexes of QBD and QBO

The ethanolic solution of a ligand (0.5 mmol) was slowly added to a 50 ml solution of metal chloride (0.5 mmol) in ethanol with continuous stirring. The reaction mixture was warmed on a water bath at 70 to 80°C for about 1 hour. The precipitate obtained was filtered, washed several times with absolute alcohol, finally with ether, and then dried over fused CaCl_2_.

## 4. RESULTS AND DISCUSSION

The complexes are microcrystalline colored powder, stable at room temperature, and are soluble in DMF and DMSO. The elemental analyses were satisfactory, show that the complexes have a ligand to metal ratio of 1 : 2 and 1 : 1, and have the general formula [ML_2_Cl_2_], where M=Co(II) or Ni(II), and [MLCl_2_], where M=Zn(II) or Cd(II); L=QBD or QBO. The molar conductance value (13.31–25.2 mhos cm^2^ mol^−1^) indicates the nonelectrolytic nature of the complexes (Table 1).

### 4.1. Electronic and reflectance spectra

The Co(II) complexes exhibit three bands in the visible region 13310–14250 cm^−1^, 14810–14988 cm^−1^, and 16425–16431 cm^−1^ pertaining to ^4^T_1g_(F) → ^4^T_2g_(F) (*ν*_1_), ^4^T_1g_(F) → ^4^A_2g_(F) (*ν*_2_), and ^4^T_1g_(F) → ^4^T_1g_(P) (*ν*_3_) transitions, respectively. These electronic spectral data were consistent with high-spin octahedral configuration around Co(II) ion [11, 12]. The electronic spectra of Ni(II) complexes of QBD and QBO show two bands in the region 12610–16260 cm^−1^and 23256–28011 cm^−1^ due to ^3^A_2g(F)_ → ^3^T_1g(F)_ (*ν*_2_) and ^3^A_2g(F)_ → ^3^T_1g(P)_ (*ν*_3_) transitions, respectively, that commensurate with octahedral stereochemistry [13]. The reflectance spectra is identical for both Zn(II) and Cd(II) complexes. The spectra of the complexes do not indicate lowest energy ^3^A_2g_→^3^T_2g_ transition. The Zn(II) complex shows two week low energy bands at ∼15155 cm^−1^ and ∼19500 cm^−1^. This is due to metal ligand charge transfer process ascribed to a charge transfer from d orbital of the metal to the π* system of the ligand [14–16]. The low-energy bands are in the position typically found for square planer configuration and may be assigned to ^2^B_1g_→^2^A_1g_ and ^2^B_1g_→^2^E_g_ transition, respectively. These complexes also exhibit more intense band at ∼37000 cm^−1^ which corresponds to intra ligand molecular charge transfer within the ligand that are assigned to n → π* transitions. The absence of d-d bands in these complexes at longer wavelength is quite reasonable since the metal ion has filled d-sub shells where d-d transitions are highly forbidden [17, 18]. By considering these data, the tetrahedral geometry has been proposed for Cd(II) [19] and square planar geometry for Zn(II) complexes [20, 21].

### 4.2. Magnetic moments

The room temperature magnetic moment value (Table 1) supports octahedral geometry for Co(II) and Ni(II) complexes. The complexes Zn(II) and Cd(II) are diamagnetic due to the unavailability of unpaired electrons [11–13, 19–21].

### 4.3. IR spectra

The IR spectra of complexes are compared with that of free ligands to determine the changes that might have taken place during complexation. The important bands and assignments of ligands and their complexes are summarized in Table 2. The results indicate that the ligands are bidentate [22–28] in nature.

The free ligand QBD exhibits strong bands at 1658 and 3330 cm^−1^ due to C=N and −NH groups, respectively. In the IR spectra of complexes, these bands shift (10–35 cm^−1^) towards the lower energies when compared with free ligands. The characteristic absorptions at 1650 and 1022 cm^−1^ in QBO were assigned to the stretching vibrations of *ν*(C=N) and *ν*(COC) groups, respectively. In the complexes, these vibrations shift to lower regions by 10–25 cm^−1^. The shift of these bands in complexes suggests the coordination of nitrogen of quinoline ring and oxygen atom of azepine ring of ligand to metal ions. The bonding of metal ion to the ligands through N, N in QBD and N, O atoms in QBO was further supported by the presence of new bands in the region 328–375 cm^−1^ due to *ν*(M−N) and *ν*(M−O) vibrations [29–31].

### 4.4. ^1^H NMR spectra

The above binding pattern is further supported by proton magnetic resonance spectral studies and chemical shift values presented in Table 2. The ^1^H NMR spectra of ligand QBD exhibits a singlet at 10.80 *δ* (s, N−H) and 8.6 *δ* (s, H−C=N). The spectra of complexes slightly changed as compared to those of corresponding ligand, and the signals appeared downfield, as expected, due to the coordination of nitrogen atoms to the metal ion [32–37]. ^1^H NMR spectrum of QBO ligand showed signals at *δ* 8.4 (s, 1H, H−C=N), 7.3–8.0 (m, 11H, Ar−H), and 2.6 (s, 3H, CH_3_). In the spectra of complexes, all signals remained in the same position except the signal of H−C=N. This is probably due to the coordinating effect of azepine oxygen atom.

## 5. BIOLOGICAL STUDIES

### 5.1. Antibacterial activity

The ligands and their Co(II), Ni(II), Cd(II) and Zn(II) complexes were tested for the in vitro antibacterial activity against *P. aerugenosa* (gram-negative) and *S. aureus* (gram-positive) bacteria by employing paper disc method [38–41]. The antibacterial activity was estimated on the basis of the size of inhibition zone formed around the paper discs on the seeded agar plates. For each concentration, the mean diameter (mm) of inhibition zone developed was calculated. The streptomycin (100 mg) was used as a standard and DMF solvent was also put to know the activity of solvent.

### 5.2. Antifungal activity

The antifungal studies of ligands and its metal complexes were tested on fungal strains namely, *C. albicans*, *A. flavus*, and *A. niger* in growth media by using Batemann poisoned-food technique [42, 43]. A known weight of the compound was dissolved in DMF in suitably labeled sterile test tubes to get a final concentration of 0.1, 0.2, and 0.3%. The sterile medium, sterile ligand, and metal complex solution were mixed under sterile conditions and allowed for solidification. The Fluconazole (100 mg) was used as a standard and DMF solvent was also put to know the activity of solvent.

The test fungi were taken as 2 mm discs from 10 days old pure colonies and placed at the center of petri dishes containing nutrient medium. The experiment was carried out in four replicates per treatment, and incubation was carried out at 30°C for 72 hours. The radial growth of colony was recorded after 96 hours of incubation and mean diameter of mycelial growth in each treatment was recorded. The average percentage inhibition was calculated on the growth media compared to the respective controls using expression [44] I = (C-T) × 100/C, where I = percentage inhibition, C = average diameter of fungal growth on the control plates, and T = average diameter of fungal growth on the tested plates (Table 3).

Antibacterial and antifungal data are presented in Table 3. This screening data clearly leads to the following conclusion.

- The complexes are slightly more toxic than their parent ligands against tested microorganisms under identical experimental conditions.
- The antimicrobial activity results indicate that the activity of Zn(II) complexes show better activity than that of Cd(II), Co(II), and Ni(II) complexes.
- The antifungal screening data clearly shows that the inhibition of fungal growth increases with increasing the concentration of complexes.

## Figures and Tables

**Table 1:** Analytical and physical data (calculated values are in parentheses).

Compound	Yield (%)	Found (Calcd) (%)					Molar conductivity mhos cm^2^ mol^−1^	Magnetic moment μ_eff_ BM	Mol.Wt. found (Calcd)
		C	H	N	M	Cl			
QBD	78	78.25	4.36	17.00	—	—	—	—	241.25
		(78.38)	(4.48)	(17.13)					(245.27)
QBO	81	78.01	4.23	11.32	—	—	—	—	240.32
		(78.06)	(4.06)	(11.38)					(246.26)
[Co(QBD)_2_Cl_2_]	85	62.14	3.18	13.44	9.16	11.38	14.8	4.41	624.56
		(62.17)	(3.23)	(13.58)	(9.53)	(11.46)			(620.39)
[Ni(QBD)_2_Cl_2_]	85	62.24	3.14	13.39	9.36	11.25	18.9	2.94	625.12
		(62.09)	(3.23)	(13.57)	(9.48)	(11.45)			(620.15)
[Cd(QBD)Cl_2_]	81	44.78	2.20	9.68	26.18	16.48	13.31	—	425.13
		(44.94)	(2.33)	(9.82)	(26.29)	(16.58)			(428.59)
[Zn(QBD)Cl_2_]	77	50.72	2.58	11.10	17.08	18.58	14.52	—	378.46
		(50.50)	(2.62)	(11.03)	(17.18)	(18.63)			(381.54)
[Co(QBO)_2_Cl_2_]	85	61.42	3.16	9.2	9.22	11.32	25.2	4.83	648.35
		(61.77)	(3.21)	(9.0)	(9.47)	(11.39)			(622.36)
[Ni(QBO)_2_Cl_2_]	83	61.49	3.05	9.00	9.24	11.25	25.7	3.01	618.25
		(61.70)	(3.20)	(9.20)	(9.40)	(11.40)			(622.12)
[Cd(QBO)Cl_2_]	80	44.59	2.34	6.42	26.08	16.41	15.84	—	426.61
		(44.73)	(2.35)	(6.52)	(26.17)	(16.51)			(429.58)
[Zn(QBO)Cl_2_]	73	50.26	2.51	7.25	17.06	18.35	16.75	—	378.76
		(50.23)	(2.63)	(7.32)	(17.09)	(18.53)			(382.6)

**Table 2:** IR and ^1^H NMR spectral data.

Compound	Infrared spectral data					^1^H NMR spectral data (δ, ppm)
	ν(C=N)	ν(NH)	ν(COC)	ν M−N	ν M−X	
QBD	1658	3330	—	—	—	10.65 (s, 1H, NH), 7.2–7.8 (m, 9H, Ar−H),
						8.4 (s, 1H, H−C=N),
QBO	1651	—	1022	—	—	8.3 (s, 1H, H−C=N),
						7.1–8.0 (m, 9H, Ar−H)
[Co(QBD)_2_Cl_2_]	1615	3320	—	440	350	10.95 (s, 1H, NH), 7.2–8.8 (m, 9H, Ar−H),
						8.4 (s, 1H, H−C=N),
[Co(QBO)_2_Cl_2_]	1615	—	995	458	368	8.3 (s, 1H, H−C=N),
						7.5–8.9 (m, 9H, Ar−H)
[Ni(QBD)_2_Cl_2_]	1620	3310	—	467	250	10.90 (s, 1H, NH), 7.2–8.8 (m, 9H, Ar−H),
						8.4 (s, 1H, H−C=N),
[Ni(QBO)_2_Cl_2_]	1631	—	996	449	264	8.3 (s, 1H, H−C=N),
						7.5–8.9 (m, 9H, Ar−H)
[Zn(QBD)Cl_2_]	1625	2990	—	428	348	10.90 (s, 1H, NH), 7.2–7.8 (m, 9H, Ar−H),
						8.4 (s, 1H, H−C=N),
[Zn(QBO)Cl_2_]	1615	—	1002	432	350	8.3 (s, 1H, H−C=N),
						7.5–8.9 (m, 9H, Ar−H)
[Cd(QBD)Cl_2_]	1610	3300	—	432	348	10.85 (s, 1H, NH), 7.2–7.8 (m, 9H, Ar−H),
						8.4 (s, 1H, H−C=N),
Cd(QBO)Cl_2_]	1610	—	998	428	360	8.3 (s, 1H, H−C=N),
						7.1–8.0 (m, 9H, Ar−H)

**Table 3:** Antimicrobial activities.

Compound	Inhibition zone of bacterial growth (mm)						Percentage inhibition of fungicidal growth								
	*P. aerugenosa*			*S. aureus*			*C. albicans*			*A. niger*			*A. flavus*		
	0.1%	0.2%	0.3%	0.1%	0.2%	0.3%	0.1%	0.2%	0.3%	0.1%	0.2%	0.3%	0.1%	0.2%	0.3%
QBD	1.2	1.6	2.8	1.4	1.8	3.0	11.2	13.8	18.3	10.3	13.4	20.2	9.8	13.1	24.2
QBO	1.1	2.0	3.0	1.3	1.6	2.9	10.3	12.9	16.9	8.8	11.9	18.6	7.8	11.8	22.3
[Zn(QBD)Cl_2_]	2.2	2.7	4.3	2.3	3.1	5.1	13.2	20.5	29.6	13.2	16.9	23.2	12.4	15.2	27.5
[Cd(QBD)Cl_2_]	1.8	2.4	3.9	2.0	2.8	4.3	12.9	18.5	25.6	12.1	16.0	22.4	11.3	14.3	26.3
[Co(QBD)_2_Cl_2_]	1.6	2.0	3.4	1.7	2.3	3.6	12.7	15.9	23.0	11.3	14.5	21.5	10.6	13.6	25.2
[Ni(QBD)_2_Cl_2_]	1.5	2.0	3.2	1.7	2.2	3.5	11.6	15.2	22.1	11.0	14.3	21.3	10.5	13.5	25.1
[Zn(QBO)Cl_2_]	1.8	3.2	4.2	2.1	2.9	4.3	12.3	19.8	28.5	11.5	14.9	21.1	10.2	14.4	25.3
[Cd(QBO)Cl_2_]	1.6	2.9	3.7	2.0	2.6	3.9	11.8	17.8	25.2	10.5	14.1	20.1	9.1	13.5	24.6
[Co(QBO)_2_Cl_2_]	1.5	2.6	3.5	1.6	2.2	3.4	11.1	15.6	22.0	9.8	13.0	19.3	8.6	12.9	23.4
[Ni(QBO)_2_Cl_2_]	1.5	2.5	3.5	1.6	2.1	3.3	11.1	14.9	21.5	9.7	12.9	19.2	8.5	12.8	23.2
Streptomycin	4.0	12.0	13.5	9.4	14.5	26.7	—	—	—	—	—	—	—	—	—
Fluconazole	—	—	—	—	—	—	36.2	55.5	70.0	36.0	40.0	65.8	37.5	48.9	75.4
DMF	+ve	+ve	+ve	+ve	+ve	+ve	+ve	+ve	+ve	+ve	+ve	+ve	+ve	+ve	+ve

DMF is used as control, +ve indicates growth of microbes.
